# Traumatic brain injury induces cell-type-specific remodelling of the proteostasis network

**DOI:** 10.1098/rstb.2025.0264

**Published:** 2026-10-01

**Authors:** Jonathan Lefkowitz, Milo Taylor, Aditya Kumar, Ruchira M. Jha, Dhivyaa Rajasundaram, Prajwal Ciryam

**Affiliations:** ^1^ Department of Neurology, University of Maryland School of Medicine, Baltimore, MD, USA; ^2^ Departments of Neurology, Barrow Neurological Institute, Phoenix, AZ, USA; ^3^ Department of Neurological Surgery, Barrow Neurological Institute, Phoenix, AZ, USA; ^4^ Department of Translational Neuroscience, Barrow Neurological Institute, Phoenix, AZ, USA; ^5^ Safar Center for Resuscitation Research, UPMC Children's Hospital of Pittsburgh, School of Medicine, University of Pittsburgh, Pittsburgh, PA, USA; ^6^ Division of Health Informatics, Department of Pediatrics, UPMC Children's Hospital of Pittsburgh, School of Medicine, University of Pittsburgh, Pittsburgh, PA, USA; ^7^ Shock Trauma Neurocritical Care, Program in Trauma, R Adams Cowley Shock Trauma Center, University of Maryland Medical Center, Baltimore, MD, USA

**Keywords:** proteostasis, traumatic brain injury, inflammation

## Abstract

Traumatic brain injury (TBI) triggers competing damage and repair processes that can result in chronic neurodegenerative protein conformational disorders. Acute alterations in proteostasis have been reported after injury, but a comprehensive understanding of changes to the proteostasis network (PN) has been limited by the complex cellular pathology of TBI. The recent enumeration of the human PN and advances in single-cell transcriptomics make it possible to delineate PN dynamics in multifactorial diseases such as TBI. Here, we leverage archival mouse and human transcriptomic data to identify cell-type-specific Sub-PNs in the brain and their alteration by TBI. We find characteristic PN gene expression profiles in many cell types and subtypes, reflecting tremendous diversity in the enrichment of specific PN processes. TBI induces a broad transcriptional remodelling of translation, the ubiquitin–proteasome system and nuclear proteostasis machinery across cell types. In addition to this shared response, numerous cell-type-specific responses occur, some of which are conserved between mouse and human, despite differences in injury mechanism and timing. Our extensive characterization of the PN transcriptome across cell type and disease reveals potentially modifiable mechanisms of PN alteration in TBI. More broadly, our results provide a framework for elucidating changes to the PN in complex diseases.

This article is part of the Theo Murphy meeting issue ‘ProteostaSys: a systems view of proteostasis’.

## 1. Introduction

Traumatic brain injury (TBI) is a leading cause of death and disability worldwide [1]. TBI results in substantial brain tissue damage resulting from the initial impact and secondary injury that occurs over the following days and weeks [2]. In the long term, victims of TBI are at risk of developing neurodegenerative protein conformational disorders [3–6], thought to arise from disruptions in proteostasis [7,8], endoplasmic reticulum (ER) stress [9,10], oxidative stress [11] and mitochondrial dysfunction [12,13]. These chronic alterations in proteostasis follow acute disruptions that occur in the hours and days after the inciting trauma [8]. For example, molecular chaperones Grp78, Hsp60 and Hsp70 are induced within hours to a day after TBI in animal models [14,15]. Increased Hsp70 expression has even been observed in the injured human brain acutely after TBI [16]. Alteration of ubiquitin gene expression and an increase in protein ubiquitination have also been observed acutely after experimental TBI [17]. Despite these and other specific findings, the field lacks a more comprehensive view of the changes in proteostasis that occur after TBI. The need for such insights is made clear by proposals for proteostasis-related therapies for TBI [18]. Unsolved problems include an understanding of the cell-type specificity of proteostasis functions and specific alterations in these functions after TBI.

Two recent developments have made it possible to achieve this comprehensive view. First, the human proteostasis network (PN) was recently defined through rigorous expert review [19,20], providing a framework for genome-wide and proteome-wide analyses. Second, advances in multi-omics approaches, particularly single-cell transcriptomics, have made it possible to determine nuanced molecular changes in complex tissues and diseases. These and related tools have been leveraged to identify neuron-specific PNs [21] and disease-specific PNs [22].

Here, we sought to identify cell-type-specific transcriptomic changes in the PN in TBI, leveraging data from two recently published datasets: (1) a well-controlled set of experiments in a mouse model of TBI and (2) more heterogeneous but clinically relevant human tissue after TBI. We identified cell-type-specific PN networks across numerous cell types in both mouse and human brain, and determined how the PN is remodelled at the transcriptional level after TBI.

## 2. Results

### (a) The proteostasis network is associated with cellular pathways relevant to traumatic brain injury

The PN is a highly interconnected set of biological processes that are involved in a wide range of cellular functions (supplementary material, figure S1). We sought to determine whether these processes are related to cellular pathways known to become altered as a result of TBI. To do so, we determined the enrichment of genes in each of the PN branches among pathways defined in the Molecular Signatures Database Hallmark gene sets [23]. The branches of the PN are closely associated with several pathways central to the response to TBI (figure 1; supplementary material, table S1). PN Regulation (PNREG) is associated with the TNF-α signalling via NF-κB pathway (odds ratio (OR): 8.8, adjusted *p*-value (*p*_adj_): 9 × 10^−4^), based largely on a set of genes that encode members of the NF-κB transcription factor family. NF-κB has been implicated as a master regulator of the inflammatory response to TBI [24,25]. Mitochondrial proteostasis (MITO) is associated with a number of genes related to oxidative phosphorylation (OR: 7.1, *p*_adj_: 8.1 × 10^−7^). Metabolic mismatch and mitochondrial dysfunction occur after TBI [13,26,27]. Extracellular proteostasis (EXTRA) is associated with both Complement (OR: 9.0, *p*_adj_: 2.2 × 10^−2^) and Coagulation (OR: 17.1, p_adj_: 4.4 × 10^−4^). This association arises from the involvement in EXTRA of genes encoding two complement proteins (C1R, C1S), thrombin (F2), the chaperone clusterin (CLU), proprotein convertase PCSK9 and the matrix-associated protein SPARC. Complement activation and coagulopathy commonly occur after TBI [28–30]. Unsurprisingly, there is an association between the autophagy–lysosome pathway (ALP) and the PI3K/AKT/mTOR pathway (OR: 3.5, p_adj_: 5.4 × 10^−6^), given the central role of mTOR in autophagy. These processes have been shown to be upregulated after TBI [31,32]. The other enriched pathways may also be associated with TBI, but this association is less well-established. Given the association of PN branches with several cellular pathways relevant to TBI, we investigated the dynamics of the PN in TBI with greater precision using single-cell genomics datasets.

**Figure 1.**
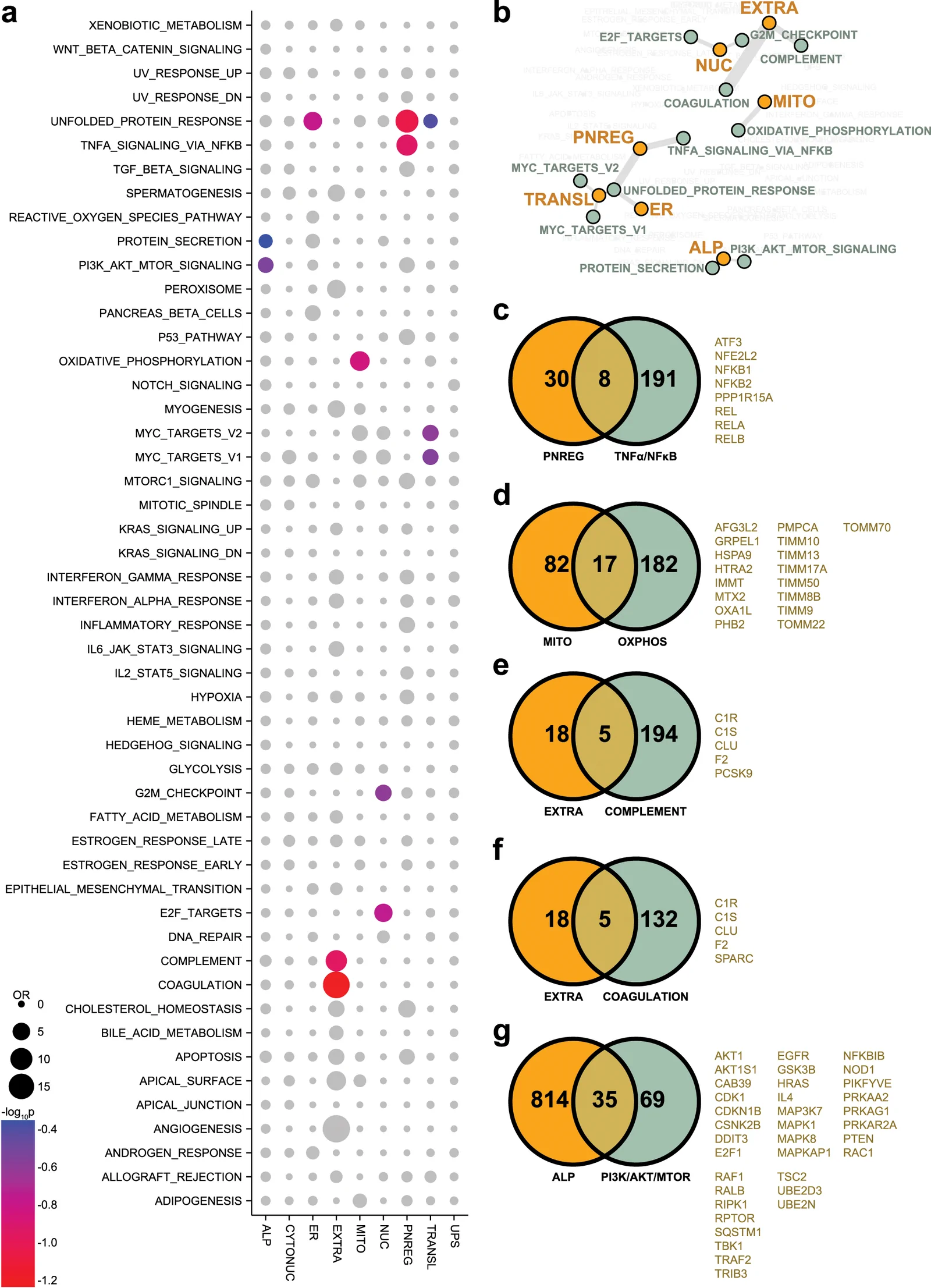
Association of the proteostasis network with other cellular pathways. (a) Dot plot showing odds ratio (OR) and –log_10_*p* (Fisher's exact test, Benjamini–Hochberg adjusted) of enrichment of PN pathways with the Molecular Signatures Database Hallmark Pathways (HP). (b) Interaction network showing connections between PN and HP. Significant pathways (*p*_adj_ < 0.05) are in orange (PN) or green (HP), with connections between PN and HP weighted by the OR. (c–g) Venn diagrams showing overlap between selected branches of the PN and pathways of the HP. ALP: Autophagy–lysosome pathway, CYTONUC: Cytonuclear proteostasis, ER: ER proteostasis, EXTRA: Extracellular proteostasis, MITO: Mitochondrial proteostasis, NUC: Nuclear proteostasis, PNREG: PN regulation, TRANSL: Translation, UPS: Ubiquitin–proteasome system.

### (b) Identifying proteostasis network gene expression in traumatic brain injury

To understand cell-type-specific changes in the expression of PN genes in TBI, we made use of two recently published single-cell/nucleus gene expression atlases of TBI: (1) controlled cortical impact (CCI) model of TBI in mice [33] and (2) single-nucleus RNA sequencing data derived from contused brain tissue of living human subjects who suffered TBI as well as deceased controls who had not died from brain-related disease [34]. Our basic strategy was to cluster PN gene expression at a single-cell/nucleus level and use this as a means to understand cell-type- and condition-specific PN gene expression using previous annotations of cell types in the data.

In the CCI model, animals undergo a focal injury to the parietal cortex on one side of their brain. The dataset we employed identified ten major cell types: neurons, microglia, oligodendrocytes, astrocytes, endothelial cells, ependymal cells, neutrophils, natural killer cells and lymphocytes (B and memory T). For our analysis, we included 52 368 cells derived from male mice assayed 24 h after CCI compared with naive mice that did not undergo injury. We performed our analysis using human homologues of mouse genes. We found that 2420 PN genes are represented across this dataset and that every cell expresses at least 48 PN genes (at read count ≥1).

In the human dataset, the authors annotated seven cell types (oligodendrocytes, oligodendrocyte precursors, microglia, astrocytes, endothelial cells, excitatory neurons and inhibitory neurons). The dataset also identified an additional small cluster of unspecified cells, which we excluded from our analysis. These data are naturally more heterogeneous in terms of sex, age, time from injury, location of injury and severity of injury, complicating the analysis. We analysed 14 724 nuclei, expressing a total of 2894 PN genes. Every cell expresses at least 130 PN genes (at read count ≥1).

### (c) A core sub-proteostasis network in traumatic brain injury

As there was expression of some PN genes in every cell, we wondered whether there was a core commonly expressed PN that might differ in control tissue and TBI. In naive mice, there are 61 PN genes that are expressed by more than half the cells of each cell type, which we describe as the Core Naive Sub-PN (figure 2a; supplementary material, table S2). Most are related to translation (TRANSL), including 45 genes encoding ribosome subunits; translation initiation and elongation genes *EIF1* and *EEF1A1*; and *NACA* and *PFDN5*, which encode proteins that play critical roles in folding nascent polypeptides. Other categories are genes for constitutively expressed chaperones (two members of the Hsp90 family (*HSP90AB1*, *HSP90B1*), *Hsc70* and *Grp78*); ubiquitins (*UBB*, *UBC*) and a ubiquitin-like protein (*FAU*); ALP-associated genes (*CALM1*, *TPT1*, *DDX5*) and extracellular proteostasis genes (*TTR*, *ITM2B*). The 44 broadly expressed genes in CCI (*Core CCI Sub-PN*) are largely similar (figure 2c), with six genes that distinguish the Core CCI Sub-PN from the Core Naive Sub-PN: *TXN*, *GNB2*, *SEM1*, *MIF*, *RPS25* and *PAFAH1B1*. *TXN* and *MIF*, which encode thioredoxin and macrophage inhibitory factor, respectively, are not only part of the PN, but also part of the inflammatory response, consistent with our observation of the interrelatedness of the PN and inflammation.

**Figure 2.**
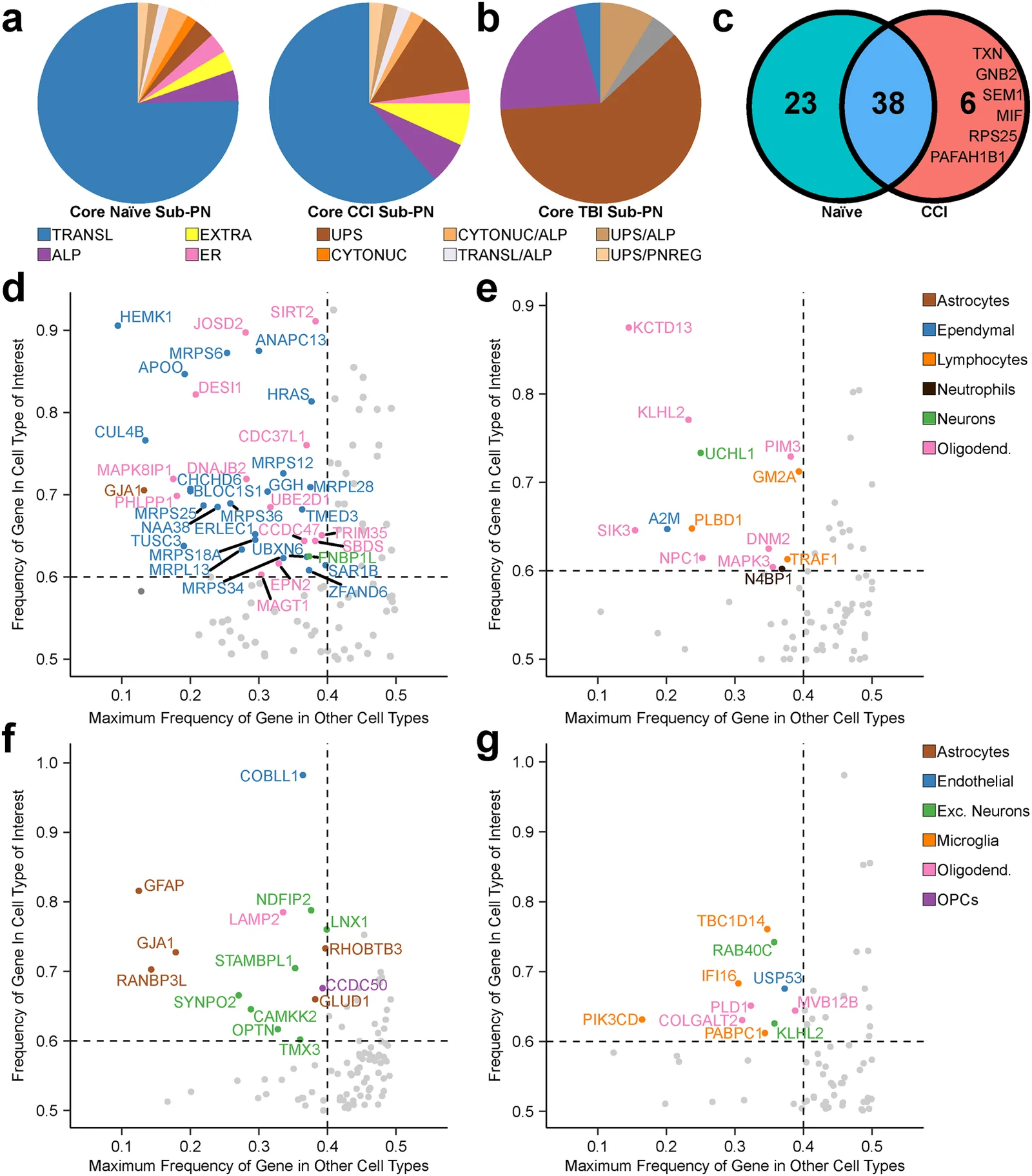
Cell-type-specific expression of the proteostasis network in mouse CCI. (a) Pie charts showing distribution of genes by branch membership in a core PN expressed in cell types in cells derived from naive (left) or CCI (right). (b) Pie chart showing distribution of genes by branch membership in the core PN expressed in cell types in cells derived from TBI. (c) Venn diagram of the overlap between the core PNs of naive and CCI, with the CCI-specific core PN genes listed. (d–g) Preferential gene expression for mouse naive (d), mouse CCI (e), human control (f) and human TBI (g) are plotted, based on frequency of gene expression in the cell type of interest and maximum frequency of gene expression among all other cell types. All genes in which frequency in cell type of interest >0.5 and maximum frequency among other cell types <0.5 are plotted. Dotted lines show thresholds of cell type of interest >0.6 other cell types <0.4, with genes meeting these thresholds labelled and highlighted by cell type.

As in mice, we derived a core PN in human tissue, but focused on the TBI cases as the controls do not reflect a truly healthy PN (figure 2b; supplementary material, table S2). The Core TBI Sub-PN is markedly different from the Core CCI Sub-PN, alhough differences between species should be interpreted with caution. For example, there are no ribosomal subunits among the Core TBI Sub-PN, unlike the Core CCI Sub-PN. Most of the core TBI Sub-PN is related to the ubiquitin–proteasome system (UPS). Almost all the genes in the Core TBI Sub-PN are shared across cell types in the control samples, as well. The two exceptions are *BTBD9* and *ITCH*, which both encode E3 ubiquitin ligases.

### (d) Identifying preferential gene expression in specific cell types

Having identified a small set of common features between cell types, our next goal was to understand how cell types differed. As an initial approach, we identified PN genes that were preferentially expressed in a single major class of cells in either naive or CCI animals, filtered to include only those genes that had overall increased expression across cell types in the condition in question (figure 2d,e; supplementary material, figure S2 and table S3). The latter constraint was to prioritize genes whose expression is more likely to be relevant in that context. This analysis revealed a small set of cell-type-preferential genes, which we then considered in greater detail.

Immune cell types (lymphocytes, microglia and neutrophils) have no preferential PN gene expression in naive animals. In the case of lymphocytes and neutrophils, this may be explained in part by the paucity of cells detected in naive animals, reflecting the relatively immune-privileged status of the brain. Microglia, on the other hand, are highly abundant, but may not have a PN-preferential signature when grouped together because of their known transcriptomic diversity [35]. Endothelial cells also do not have preferentially expressed genes based on these cutoffs. By contrast, the ALP-associated genes *FNBPL1* and *GJA1* are preferentially expressed in neurons and astrocytes, respectively. Oligodendrocytes preferentially express 13 genes. Ependymal cells preferentially express 23 genes, far more than any other cell type, including eight genes encoding ribosomal subunits. Consistent with the secretory role of the ependyma, these cells preferentially express genes involved in endoplasmic reticulum proteostasis (ER), including *ERLEC1*, *SAR1B* and *TMED3*. This reflects the distinct transcriptional profile of this important but understudied cell population.

Notably, CCI induces more preferential PN gene expression in systemic immune cells that enter the brain, but not in microglia (figure 2d,e; supplementary material, figure S2b). The latter is again probably a function of the tremendous diversity of microglia. Neutrophils specifically express *N4BP1*, which encodes a multi-functional protein that inhibits E3 ubiquitin ligase, regulates NF-κB and has endoribonuclease activity [36,37]. Lymphocytes preferentially express *TRAF1*, *PLBD1* and *GM2A* in the CCI condition. The cell-type preferential PN response is remodelled in other cell types. It is striking that only one PN gene is preferentially expressed in ependymal cells after CCI: *A2M*, which encodes alpha-2-macroglobulin. As expected, the known TBI neuronal injury marker UCHL1 [38] has neuron-preferential expression after CCI, highlighting the prominent role of the PN in well-established TBI-related processes. No genes overlap between naive and CCI tissue for preferential expression in oligodendrocytes, whereas several genes are unique to CCI, including: *DNM2*, *KCTD13*, *KLHL2*, *MAPK3*, *NPC1*, *PACS2*, *PIM3* and *SIK3*.

We undertook a similar analysis for human tissue (figure 2f,g; supplementary material, figure S3 and table S3). There is little overlap between genes preferentially expressed in particular cell types in human and mouse tissues. Systemic immune cells are not represented in the human dataset, but microglia are. While no genes are preferentially expressed in microglia from control tissue, in TBI-derived tissue, inflammatory genes *IFI16* and *PIK3CD* are preferentially expressed in microglia, as are the genes *PABPC1* and *TBC1D14*. Human astrocytes in control tissue, like those in naive tissue, preferentially express *GJA1*. These cells also preferentially express *GFAP*, *GLUD1*, *RANBP3L* and *RHOBTB3*. No genes are preferentially expressed in TBI-derived astrocytes. Oligodendrocytes from control tissue preferentially express the ALP-associated gene *LAMP2*. The ALP genes *PLD1* and *MVB12B* are preferentially expressed in oligodendrocytes derived from TBI tissue, along with the endoplasmic reticulum proteostasis (ER) gene *COLGALT2*. Oligodendrocyte precursor cells (OPCs) preferentially express *CCDC50* in control but not TBI tissue. Though interneurons do not have any preferentially expressed genes, excitatory neurons preferentially express a number of genes. Notably, *KLHL2* is preferentially expressed in excitatory neurons of TBI tissue, as it is in oligodendrocytes after CCI. *KLHL2* upregulation has been observed in CA1 hippocampal neurons in a mouse model of mild TBI [39].

### (e) Cell-type- and disease-specific mouse sub-proteostasis networks

To better define cell-type-specific Mouse Sub-PNs (MPNs) in control and injured mouse brain, we reclustered the mouse single-cell RNA sequencing data using gene expression only from PN genes (figure 3; supplementary material, table S4). Our iterative clustering approach prioritized cluster stability and specificity of cell types for individual clusters, as described in §4. Remarkably, this approach achieves specificity for individual cellular subtypes (as defined in the original analysis by Jha *et al.* [33]) despite excluding over 80% of the genes in the full transcriptome dataset (figure 3a,b). The top individual genes that contribute to these clusters are shown in figure 3d. Naive and CCI tissues cluster separately based on PN expression, with high representation of MPN Cluster 1 most notably characterizing naive tissue (figure 3c,f). This indicates that many individual cellular subtypes express distinct PN profiles (figure 3f,g).

**Figure 3.**
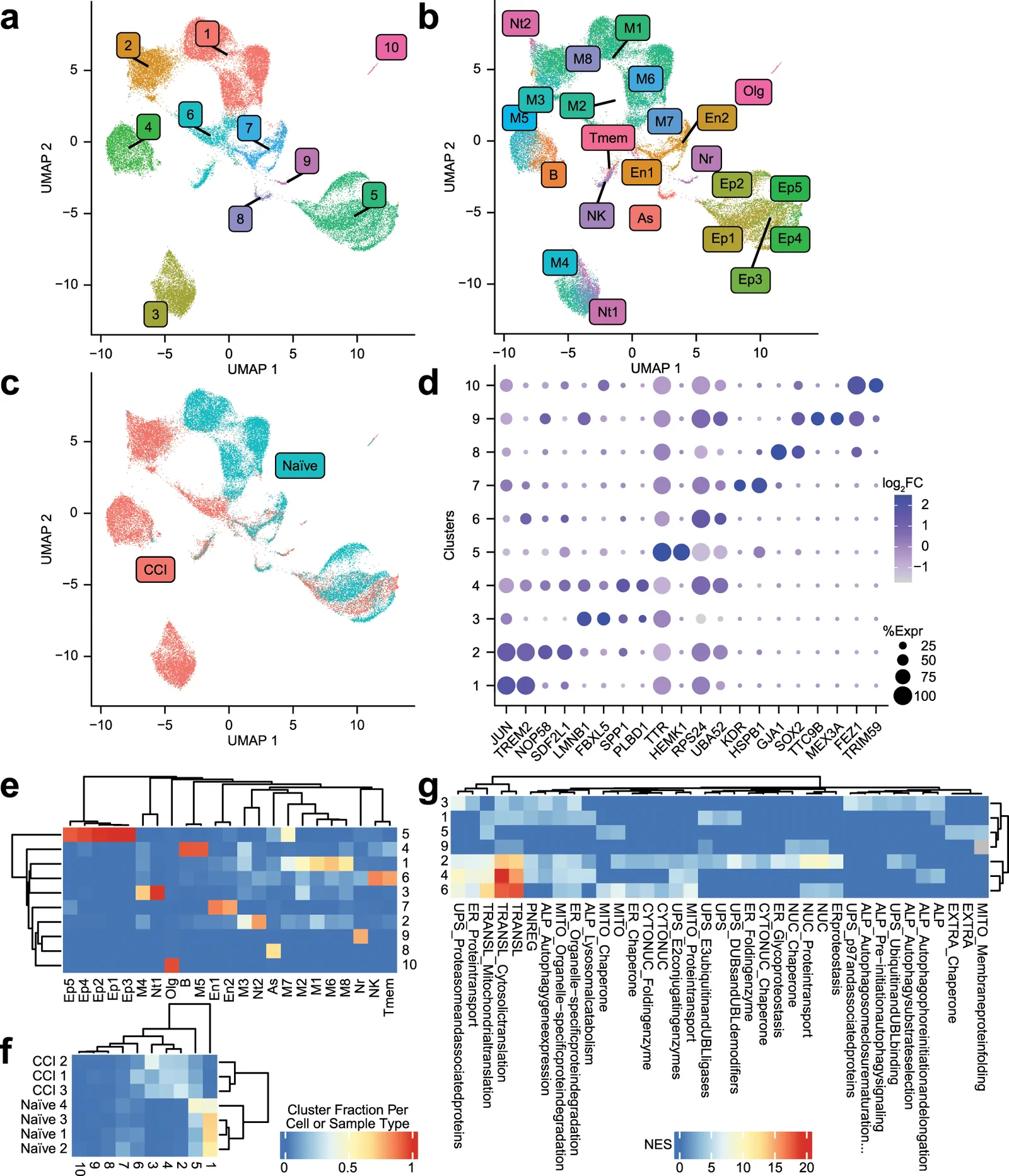
Cell-type-specific PN sub-networks in mouse CCI. Reclustering based only on PN genes (dimensions = 50, resolution = 0.5) labelled by (a) clusters, (b) cell sub-types and (c) treatment condition. (d) Dot plot showing a subset of markers associated with one or more clusters. Fraction of cells in each (e) cell subtype or (f) sample present in each cluster. (g) Normalized enrichment score (NES) for each cluster derived from gene set enrichment analysis (GSEA) using the R library for fast gene set enrichment analysis (fgsea) (positive enrichment only). Dark blue boxes denote NES = 0 or fgsea *p*_adj_ ≥ 0.05. Grey boxes denote that fgsea could not compute NES. As: astrocytes, B: B lymphocytes, En: endothelial cells, Ep: ependymal cells, M: microglia, NK: natural killer cells, Nr: neurons, Nt: neutrophils, Olg: oligodendrocytes, Tmem: memory T lymphocytes.

Some cell types/subtypes associate very closely with one MPN cluster—ependymal subtypes with MPN Cluster 5 or oligodendrocytes (Olg) with MPN Cluster 11. On the other hand, several microglial subtypes (Microglia-1, Microglia-2, Microglia-6 and Microglia-8) fall predominantly into one cluster (MPN Cluster 1), but also have substantial representation in other clusters (MPN Clusters 2 and 6, among others). It should be noted that cell subtype annotations are derived from Jha *et al.* [33]; the Microglia-1 and Microglia-2 nomenclature here does not correspond to canonical M1 and M2 phenotypes. We next performed gene set enrichment analysis (GSEA) to identify enrichment in expression of genes in PN branches and classes. Our intent in doing so was to determine the aspects of the PN that contribute to each cluster. To achieve this, we applied the PN-derived clusters to the original dataset including all genes. In figure 3g, the normalized enrichment scores for pathways are shown for each PN cluster that was significantly enriched (*p*_adj_ < 0.05) in at least one pathway. Among the seven clusters that have some PN enrichment, every cluster is enriched in pathways related to TRANSL. In the case of TRANSL, this reflects an overall high degree of similarity in the enriched genes (overlap ranging from 32% to 91%, with a median of 57%), although some clusters have starkly different profiles of enriched genes. All but one cluster (MPN Cluster 5) is enriched in UPS pathways. The UPS genes enriched in each cluster bear little resemblance to each other (overlap ranging from 17% to 53%, with a median of 25%), pointing to cluster- and therefore cell-subtype-specific UPS profiles. MPN Cluster 5, associated predominantly with ependymal cells, is enriched in TRANSL, MITO and EXTRA.

Based on their extent of pathway enrichment, we classified PN clusters into three groups: PN Strongly Enriched (MPN Clusters 2, 4 and 6); PN Moderately Enriched (PN Clusters 1, 3, 5 and 9); and PN Unenriched (MPN Clusters 7, 8 and 10). There is most clearly an enrichment in TRANSL pathways, with the strength of the enrichment distinguishing the PN Strongly Enriched from the PN Moderately Enriched clusters. The PN Strongly Enriched clusters also have extensive enrichment in pathways related to the UPS, ER, cytonuclear-proteostasis (CYTONUC) and MITO, most of which are absent from or diminished in the PN Moderately Enriched Clusters. The Moderately Enriched clusters are not uniform in their pathway enrichment. MPN Cluster 3 has broader pathway enrichment than the others, while MPN Clusters 5 and 9 have the narrowest.

Some cell types, such as neurons and ependymal cells, associate clearly with the Moderately Enriched group (MPN Clusters 9 and 5, respectively). In other cases, the delineation is less clear. For example, one subtype of neutrophils (Neutrophils-1, associated with MPN Cluster 3) is related to the Moderately Enriched group, while the other (Neutrophils-2, associated with MPN Cluster 2) is related to the PN Strongly Enriched group. Microglia-4 is fairly specific to the Moderately Enriched group (MPN Cluster 3). By contrast, Microglia-1, Microglia-2, Microglia-6 and Microglia-8 are divided between Moderately Enriched clusters (MPN Clusters 1 and 3) and Strongly Enriched Clusters (MPN Clusters 2 and 6). There is diversity among the Moderately Enriched clusters. MPN Clusters 1 and 3 are enriched in a wide range of proteostasis functions, although MPN Cluster 3 is particularly enriched in ALP-related pathways. Distinguishing the two is the enrichment of MPN Cluster 3 but not MPN Cluster 1 in UPS. MPN Cluster 5 is enriched in TRANSL, MITO and EXTRA pathways; and MPN Cluster 10 is enriched inCYTONUC pathways. The Unenriched group is associated with astrocytes, endothelial cells and oligodendrocytes. For astrocytes and endothelial cells, this is consistent with the very few cell-type-preferential genes expressed by these cell types. On the other hand, 21 PN genes are preferentially expressed by oligodendrocytes, of which 17 genes are associated with UPS or ALP (supplementary material, table S3). Despite this, the PN-wide transcriptional profile does not suggest enrichment with any pathway based on GSEA.

### (f) Cell-type- and disease-specific human sub-proteostasis networks

In human samples, PN-based clustering (HPNs) (supplementary material, table S4) was robust in identifying astrocytes, endothelial cells, microglia, oligodendrocytes and oligodendrocyte precursors (OPCs), but was less successful at revealing specific neuronal PN profiles (figure 4). We focused on the five clusters (HPN Clusters 2, 5, 10, 13 and 17) that mapped specifically onto all cell types except for neurons (figure 4e). HPN Cluster 2 (associated with oligodendrocytes) is broadly enriched in expression of PN pathways related to TRANSL, UPS, MITO, ER, PNREG and ALP (figure 4g). This broad-based enrichment of PN pathways in human oligodendrocytes differs markedly from what we observed in mouse oligodendrocytes. HPN Cluster 13 (microglia) is associated with TRANSL, ALP, PNREG and UPS. At a branch level, this is similar to patterns observed in mouse microglia. HPN Cluster 13 shares many enriched pathways with HPN Cluster 17 (endothelial cells). There are also several notable differences between these clusters. HPN Cluster 13 is enriched in the UPS class ubiquitin and UBL binding, while HPN Cluster 17 is enriched in the ER class folding enzyme. Even their shared branches belie some diversity in response. For example, the ALP response in these two clusters is overlapping, but not identical. While they share enrichment in ALP classes related to autophagy gene expression and autophagophore initiation and elongation, they differ in other classes. HPN Cluster 13 is enriched in lysosomal catabolism and pre-initiation autophagy signalling, while Cluster 17 is enriched in chaperone-mediated autophagy. While HPN Clusters 13 and 17 both are enriched in cytosolic translation, only HPN Cluster 13 is enriched in mitochondrial translation. HPN Clusters 5 and 10, associated with OPCs and astrocytes, respectively, are not enriched in any PN branches or classes. Control samples cluster together based on HPN cluster membership, but TBI samples are divided into two separate groups. These two TBI groups are distinguished by HPN Cluster 1 (weakly associated with interneurons) versus HPN Cluster 2 expression (associated with oligodendrocytes).

**Figure 4.**
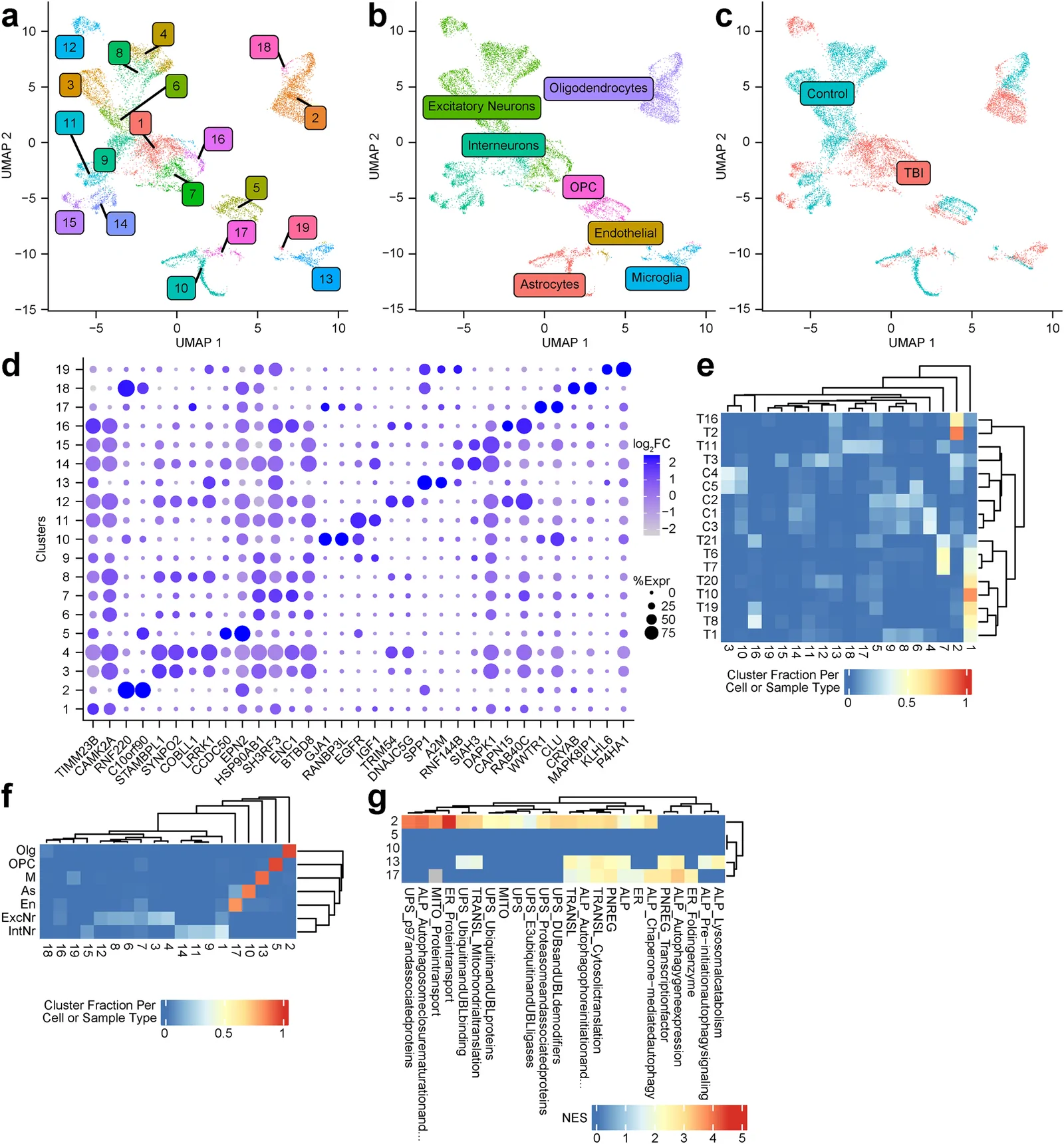
Cell-type-specific PN sub-networks in human TBI. Reclustering based only on PN genes (dimensions = 50, resolution = 1) labelled by (a) clusters, (b) cell sub-types and (c) treatment condition. (d) Dot plot showing a subset of markers associated with one or more clusters. Fraction of cells in each (e) cell subtype or (f) sample present in each cluster. (g) NES for each cluster derived from GSEA using fgsea (positive enrichment only). Dark blue boxes denote NES = 0 or fgsea *p*_adj_ ≥ 0.05. As: Astrocytes, En: Endothelial cells, ExcNr: Excitatory neurons, IntNr: Interneurons, M: Microglia, Olg: Oligodendrocytes, OPC: Oligodendrocyte precursor cells.

### (g) Remodelling of ubiquitin–proteasome system and subcellular-proteostasis networks after traumatic brain injury

We next sought to understand how TBI affects the PN. To do so, we performed pseudobulk differential gene expression analysis on a cluster and cell-subtype-specific basis, comparing control and injury samples (figure 5). Only clusters/cell types for which there were a sufficient number of cells across samples were included (see §4 ); cell counts contributing to the analysis are shown in tables S5 and S6, supplementary material.

In mice, TRANSL (cytosolic translation) and UPS (proteaosome and associated proteins) are upregulated in CCI across most MPN clusters and cell types (figure 5a). Although classes associated with ALP, CYTONUC, ER, MITO, and NUC are also broadly differentially expressed in CCI across clusters, analysis of cell types shows greater specificity (figure 5b).

Two clear groups are apparent in the cluster of cell types. There is a group of cell subtypes (B lymphocytes, endothelial cells, Microglia-1/3/6) that is robustly upregulated in subcellular-proteostasis pathways (CYTONUC, MITO, ER), whereas the other group (astrocytes, Endothelial cells-1, Epithelial cells 2/3, Microglia-2, NK cells, neurons) is not. The former group, upregulated in subcellular-proteostasis pathways, is largely downregulated in E3 ubiquitin and UBL ligases. The cell types not upregulated in subcellular proteostasis are largely downregulated in ALP lysosomal catabolism. Among the latter, ependymal cells are specifically downregulated in ER and MITO pathways, whereas other brain-resident cells (astrocytes, Microglia-2, neurons) merely lack significant regulation of most of these pathways. This is validated in the cluster-based analysis (figure 5a). The use of both cell-type-based and cluster-based analysis also provides some insights into clusters or cell types excluded for want of data. For example, the M4 subtype is poorly represented and so excluded from the pseudobulk analysis, but based on its MPN Cluster 4 profile, we can infer that it differs from other microglia in being upregulated in ALP pathways (figure 5b).

**Figure 5.**
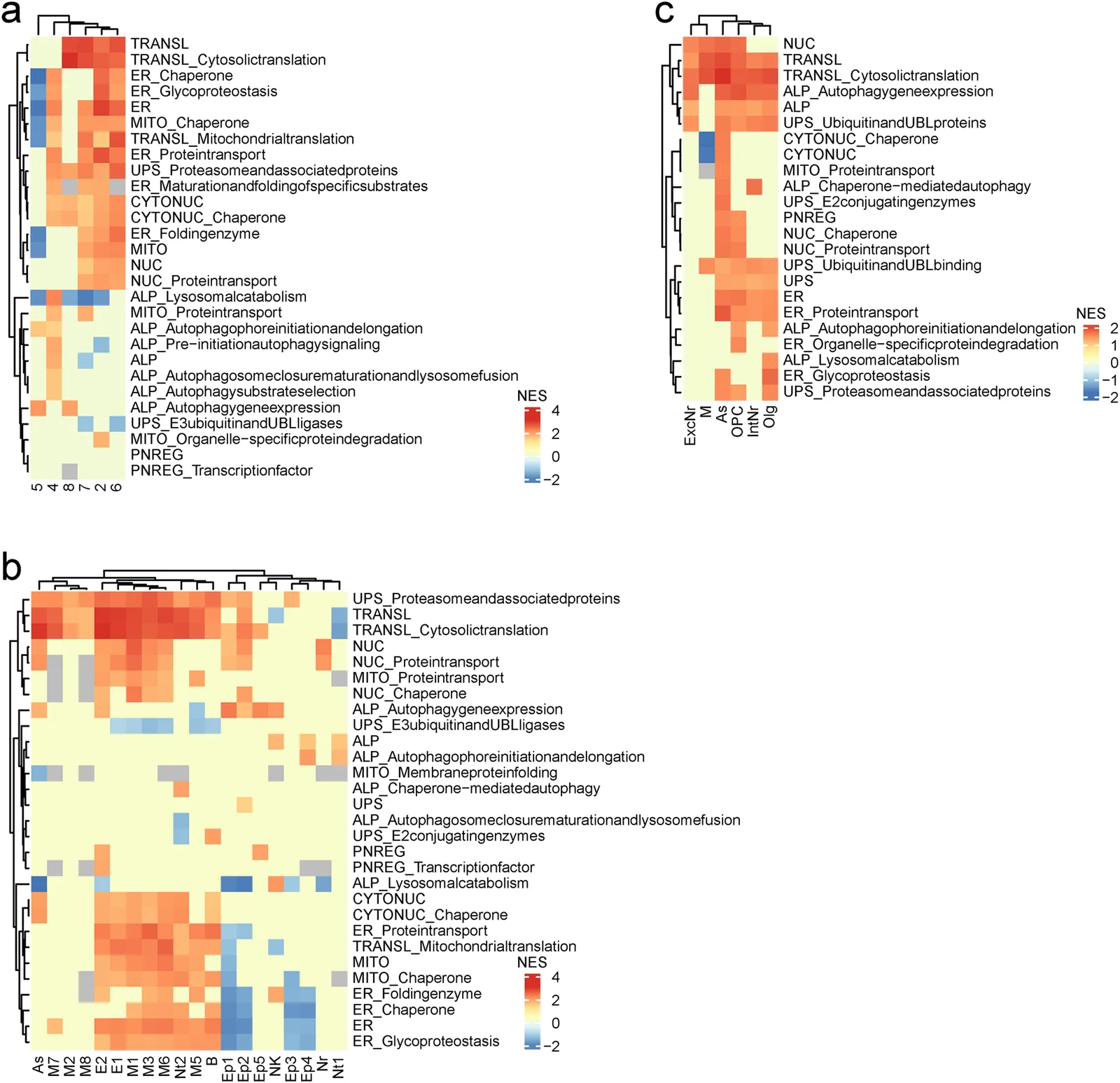
PN alterations in response to TBI. GSEA (positive and negative enrichment) showing PN branch and class enrichment in genes differentially expressed in mouse CCI by (a) cluster or (b) cell subtype. (c) Enrichment in human TBI by cell subtype. Grey boxes denote incalculable NES.

We conducted a similar analysis on human samples (figure 5c). However, we did not perform cluster-based analysis because many samples had very few cells in some clusters. We again found widespread upregulation of TRANSL, UPS and NUC, as well as ALP. Notably, microglia are not upregulated in ALP, similar to most mouse microglial subtypes. By contrast, astrocytes are upregulated in ALP, as well as subcellular-proteostasis pathways, after CCI in human but not in mouse tissue.

### (h) Diversity of the proteostasis network in mouse microglia

Microglia are the most prevalent cell type in the mouse dataset and they possess tremendous diversity in their transcriptomes in general and their PN transcriptomes in specific. We therefore sought to use these data as a means to assess the diversity of the PN in microglia. A similar analysis was not possible in human samples, in which only 638 microglia were present. Based on our clustering analysis, we observe distinct Sub-PNs associated with various subtypes of microglia, as defined by Jha *et al.* [33]. MPN Clusters 1–5 are most closely associated with microglia (figure 3e; supplementary material, table S4). At least one-third of the cells in every microglial subtype are members of a single one of these MPN clusters. The other MPN clusters have very limited membership of microglia (<20%).

Cells in MPN Cluster 1 constitute the majority of Microglia-1 (67%), Microglia-2 (50%), Microglia-6 (72%) and Microglia-8 (61%). This is a Moderately Enriched cluster notable for enrichment in TRANSL, ALP, UPS, PNREG, MITO and ER pathways (figure 3g; supplementary material, table S4). The plurality of Microglia-7 (48%) is part of MPN Cluster 5, an otherwise ependymal-specific cluster that has a narrower spectrum of PN enrichment.

The other microglial subtypes are intriguing in that their PN expression profiles more closely group with specific systemically derived immune cell subtypes: Microglia-3 is associated with Neutrophils-2, Microglia-4 with Neutrophils-1 and Microglia-5 with B lymphocytes. These microglial subtypes are associated with MPN Clusters 2 (40%), 3 (71%) and 4 (92%), respectively. These clusters are strongly associated with CCI, unlike MPN Cluster 1, which is strongly associated with naive animals (figure 3f). MPN Cluster 5 does not have a clear condition-specific association, which may be related to the substantial additional membership of ependymal cells in this cluster. While MPN Clusters 2, 3 and 4 do not clearly group separately from Clusters 1 and 5, these groups of clusters are different in another respect. Based on the number of pathways enriched, Clusters 2, 3 and 4 have a broader spectrum of PN enrichment than do Clusters 1 and 5. This may reflect either a broader regulation of specific cell types, or the inherent diversity of clusters that include microglia along with another cell type.

To assess this directly, we performed GSEA for PN branches and classes on the microglial subtypes (supplementary material, figure S4). It is clear that TRANSL is enriched across all microglia subtypes (except Microglia-7, which has no specific enrichment), although it is the most pronounced in Microglia-2, Microglia-3 and Microglia-5. The other clear division between subtypes is that Microglia-6, Microglia-7 and Microglia-8 are minimally enriched in UPS and ALP in contrast to the robust enrichment in the other microglial subtypes. These are comparatively low prevalence subtypes, potentially limiting our ability to identify significant changes. It is notable that the clustering of these pathways does not map in an obvious way onto associations with peripheral immune cell clusters or with disease phenotype, suggesting that both the naie and injured conditions possess a complex ecosystem of microglia with varying proteostasis capacities.

## 3. Discussion

Here, we have undertaken a comprehensive analysis of the PN in the context of TBI, using two recently published atlases of the brain transcriptome in mouse CCI and human TBI. We focused our analysis on TBI because it is a common disease with few treatments in which alterations in proteostasis have been reported but are poorly understood. The extraordinary complexity of this disease has made detailed characterization of the PN particularly challenging. Our work contributes to a growing body of evidence that suggests that the PN is specific to cell types and states [40], despite the essential and highly conserved nature of many PN components. We report that the expressed PN transcriptome in the brain is cell-type-specific and substantially remodelled after TBI.

The PN has long been thought to manifest in a cell-type-specific manner. Distinct cellular functions necessitate distinct expressed proteomes, each with its own proteostasis requirements to maintain stability [40]. Despite this conceptual framework, the details of cell-type-specific PNs have been elusive, especially in complex organs like the brain. This problem has become more tractable since the enumeration of the human PN and the development of methods for the detailed molecular characterization of large numbers of individual cells.

For the PN, as is true of the transcriptome as a whole [35,41], detailed analysis reveals that cell-type expression is more heterogeneous than previously thought. Nevertheless, themes emerge. In general, enrichment is more common in TRANSL, ALP, UPS, MITO and ER than in other PN branches. This analysis is based on our GSEA approach. It should be noted that the shared feature space between the cluster-defining PN genes and the PN-defined GSEA pathways probably influences downstream enrichment, but this was an intentional approach used to identify the PN features that contribute to each cluster.

PN gene expression alone contains a remarkable amount of the information to specify cell type, emphasizing the distinct Sub-PNs across cell types. In human tissue, it is notable that oligodendrocytes have a qualitatively and quantitatively distinct PN profile from microglia and endothelial cells, whose PN profiles are quite similar. PN expression profiles may reflect the specialized functions of their associated cell types. For example, mouse and human microglia are enriched in ALP pathways that are closely related to these cells’ phagocytic functions. Extracellular chaperones, such as alpha-2-macroglobulin, arise as a key node in the PN of ependymal cells. Consistent with this, the ependymal MPN Cluster 5 is associated with EXTRA, consistent with a highly secretory tissue that interfaces with extracellular cerebrospinal fluid in the cerebral ventricles. MPN Cluster 10 is associated with neurons; it may be that the complexities of intracellular trafficking and synaptic function increase cytonuclear–proteostasis requirements.

Disease has a focusing effect on the PN. There is broad-based upregulation across most cell-types of PN classes associated with TRANSL, UPS and NUC. Ependymal cells are a potential example of this focusing effect as they preferentially express 23 different genes in the naive state, but just one—*A2M*—after CCI. This may arise from differences in cell abundance and global changes in gene transcription associated with injury. A more intriguing possibility is that the PN is being reoriented toward specific functions necessary for the disease response. *A2M* encodes alpha-2-macroglobulin, which is secreted by ependymal cells and limits inflammation by inhibiting proteolysis and acting as a molecular chaperone [42].

Another key observation is that cell types are differentiated by their enrichment in components of the PN, but that these differences do not predict the changes that occur after injury. For example, ALP classes are enriched across most microglial subtypes (supplementary material, figure S4), but these classes are largely not upregulated in response to injury (figure 5a,b). This is consistent with observations that at 1 d post-CCI, M1 microglia outnumber M2 microglia [43]. As the PN is probably to be dynamic, it will be important to establish the time course of PN changes in future investigations.

Many transcriptional features appear starkly different between mouse and human. For example, both oligodendrocytes and neurons have much broader PN upregulation in response to TBI in human than mouse tissue, while the opposite is true of microglia (figure 5). Microglia are also apparently far more diverse in mouse tissue. Species-dependent differences would not be surprising [35,44], but they do raise questions about the limitations of rodent models for the study of complex neurological disease. However, cross-species comparisons in the datasets that we analysed are limited by differing age, injury mechanism and tissue processing between species. In control samples, some cross-species differences may arise from systematic differences in relative age and the fact that the human controls are not truly healthy. Another source of variability is the prevalence of recovered cells: more oligodendrocytes and neurons in human samples compared with more microglia in mouse. This may be related to biases introduced by tissue processing for single-cell (mouse) versus single-nucleus (human) experiments. Therefore, the cross-species observations we make must be interpreted as somewhat speculative.

Despite the advantages of our approach, it has some limitations. The sample sizes of the data we used are small, limiting the statistical power of our analysis. This may be a particular limitation of the human samples, which are also highly heterogeneous. They vary in age, sex, injury mechanism, anatomical location of resected tissue and time from injury to resection. In addition, the controls and the disease tissue have certain systematic differences, most notably in the source of tissue (post-mortem vs. surgical) and in age (the controls are substantially older). Therefore, differences in the PN between controls and TBI samples may be confounded by age-related changes. A more fundamental limitation of our study is that it is RNA-based instead of protein-based, and therefore is an indirect measure of the status of the PN. These approaches do not measure actual changes in concentrations of PN proteins or their functional effects on protein synthesis, folding and degradation. A comprehensive understanding of proteostasis in complex living tissue will require methods that enable cell-type- and disease-specific functional assays of the PN. Finally, we restricted our analysis to a single timepoint, but additional longitudinal data are available and would be amenable to the approaches we have established here.

Our results exist within the context of important recent efforts to delineate cell-type- and disease-specific Sub-PNs. Pechmann analysed observed that chaperone and ubiquitin ligase gene expression can differentiate excitatory neurons, inhibitory neurons and glial cells [21]. Consistent with this, we observe that four out of the seven genes preferentially expressed in excitatory neurons in control tissue in our analysis are associated with UPS. These include genes encoding the ubiquitin ligase LNX1; the HECT-domain E3 ubiquitin ligase activator NDFIP2; the ubiquitin-binding protein OPTN and the deubiquitinase STAMBPL1 (supplementary material, table S3). However, we do not observe branch- or class-level enrichment in neurons, which may be a function of the fewer number of cells included in our analysis. Our study complements Pechmann's in that we differentiate various types of non-neuronal brain cells, showing tremendous diversity in the cell-type-specific Sub-PNs of these cells. In addition, we identify disease-related changes, which notably include two E3 ubiquitin ligases specific to the CCI Core Sub-PN. Consistent with the observation that the enriched PN is not predictive of the upregulated PN, neither UPS-related classes nor chaperone-related classes are upregulated in human excitatory neurons after TBI or mouse neurons after CCI (figure 5).

Ours is not the first effort to employ the human proteostasis network [19,20] to characterize human disease. Lim and Vendruscolo [22] classified diseases based on patterns of PN component representation in the pathways of 32 diseases from seven disease groups. They found three general patterns of proteostasis perturbation that differentiated between disease states: ALP^+^UPS^+^ER^-^ (cancer), ALP^+^UPS^+^ER^+^ (neurodegeneration) and ALP^+^UPS^-^ER^+^ (other). Some similarities to and differences from these patterns exist in TBI (figure 5c). That said, some aspects of the scheme proposed by Lim and Vendruscolo are apparent at a cell-type-specific level. For instance, excitatory neurons exhibit an ALP^+^UPS^+^ER^−^ pattern similar to cancers, while several other cell types exhibit an ALP^+^UPS^+^ER^+^ pattern similar to neurodegeneration. However, TRANSL is notably underrepresented among perturbations in the diseases analysed by Lim and Vendruscolo, but is prominently dysregulated across most cell types in both mouse and human TBI (figure 5). We also observed cell-type-specific changes in a variety of other PN branches that show only modest involvement in their analysis, including MITO, NUC and CYTONUC. As the post-traumatic inflammatory state can lead to both secondary injury and necrosis, there are probably inflammatory stages that progress sequentially after TBI that differ from the chronic processes observed in cancer, autoimmune disease and neurodegeneration by Lim and Vendruscolo. More generally, our results point to the value of incorporating cell-type-specific data into such bioinformatic disease analyses. It may be that the PN patterns observed by Lim and Vendruscolo occur simultaneously in individual diseases, manifesting in specific cell types based on both the physiological and pathological roles they play. In TBI, additional multi-omic data may facilitate the development of models for cell-type-specific PN perturbation over time.

In summary, we have undertaken a comprehensive transcriptomic analysis of PN regulation in TBI, a prevalent disease known to involve PN dysregulation but understudied in this context. Our analysis reveals tremendous variation in the PN among the diverse cell types of the brain. We have found that TBI induces a core transcriptomic response in the PN across cell types and additional cell-type-specific responses that may be tailored to cells’ particular functional needs. These results provide an extensive characterization of the PN in TBI that set the stage for defining PN changes across time, sex, disease severity and brain region. Moreover, we present a framework for using single-cell transcriptomic data in conjunction with the human proteostasis network to elucidate cell-type- and disease-specific PN responses. This approach is widely applicable to diseases that implicate the PN. In the future, we anticipate that these approaches will result in new mechanistic insights about complex human disease that recapitulate the vast distribution of cellular responses.

## 4. Methods

### (a) Proteostasis network and Hallmark Pathway association

Molecular Signatures Database Hallmark Pathways [23] were downloaded and their overlap with each branch of the PN was determined. Fisher's exact test was used to compute an enrichment odds ratio and *p*-value. Adjustment for multiple hypotheses using the Benjamini–Hochberg method (*p*_adj_ = *q*-value) was performed, treating tests for each branch of the PN as a separate family.

### (b) Proteostasis network interconnectedness

The Jaccard Index (number of genes in the intersection between sets divided by the number of genes in the union of sets), expressed as a percentage, was calculated as an estimate of the interconnectedness of PN branches. Fisher's exact test was also performed. Adjustment for multiple hypotheses was performed using the Holm–Bonferroni method.

### (c) Transcriptomic data pre-processing

Datasets were downloaded and analysed using Seurat v. 5.4.0 [45], filtered for high-quality cells. Genes that were expressed in fewer than ten cells per sample were excluded, and cells that expressed fewer than 200 features were excluded. For the mouse dataset, biomaRt v.2.60.1 [46] was used to obtain gene mapping of mouse to human genes, and all mouse genes that could be unambiguously mapped were converted to human genes. For the human dataset, available data had already been pre-filtered to remove low-quality cells. For human data, cells that were annotated as unspecified were excluded from analysis. Data normalization was performed using the Seurat NormalizeData() function. Data were scaled using all PN genes with the Seurat ScaleData() function. Principal component analysis (PCA) was performed using all PN genes as features.

### (d) Data clustering

Our overall goal in clustering was to arrive at a set of robust clusters in which the cells of each pre-defined cell type (as described in the original dataset) were assigned to a single cluster to the greatest extent possible. To achieve this, a shared nearest neighbour graph was constructed with the Seurat FindNeighbors() function, separately using 20, 30 or 50 dimensions. Clusters (‘original clusters’) were then identified using the Seurat FindClusters() function at resolutions from 0.1 to 2.0. Clustering performance was evaluated by visual inspection of the clustering tree produced by clustree v. 0.5.1 [47]. In addition, the clusters were evaluated quantitatively using MRTree v. 0.0.0.9000 [48] to determine the adjusted Rand Index as a function of resolution and number of clusters, as well as the Adjusted Multiresolution Rand Index as a function of number of clusters. These metrics were used to select a subset of resolutions at each level of dimensions. For this set of clusters, the Seurat FindAllMarkers function was used to identify markers of each cluster with *p*_adj_ < 0.05, average log_2_FC ≥ 0.585 and pct.1 ≥ 0.5. The markers of each cluster were compared against those of each other cluster, and those cluster pairs with Jaccard Index ≥33.3% were merged (‘merged clusters’). To evaluate cluster performance, the fraction of each cell type/subtype assigned to each cluster was determined. In some cases, these merged clusters were not strongly associated with any cell type, contributing to less than 20% of any cell type. These poor-performing clusters invariably had not been subject to a merge in the initial re-clustering. Original clusters were identified whose Jaccard Index with the poor-performing cluster were ≥20%. Merged clusters to which these original clusters belonged were evaluated as potential candidates into which to merge the poor-performing cluster. For each candidate merged cluster, the Jaccard Index was computed as above between the poor-performing cluster and each original cluster in that candidate merged cluster. The candidate merged cluster that had the highest average Jaccard Index with the poor-performing cluster was selected and the poor-performing cluster was merged into it. In the final analysis, merged clusters in mouse were derived from dimensions = 50 and resolution = 0.5. Merged clusters in human were derived from dimensions = 50 and resolution = 1.

### (e) Preferential gene expression

We define a gene as being expressed preferentially in a particular condition if ≥60% of cells of a given cell type and ≤40% of cells of every other cell type express that gene in that condition and the average log_2_FC > 0 for all cells in that condition versus the other (e.g. naive vs. CCI).

### (f) Pseudobulk analysis

Pseudobulk analysis was performed by grouping expression data by cell type and condition using the AggregateExpression() function in Seurat. DESeq2 was then used to perform differential gene expression using disease condition as the only model variable.

### (g) Gene set enrichment analysis

For GSEA analysis, a PN-specific GSEA database was generated using PN branches and classes. The PN-defined clusters were projected onto the transcriptome dataset that included all genes, so that PN enrichment could be assessed. GSEA with fgsea v. 1.30.0 [49] was performed for (1) clusters and cell types across all cells based on average log_2_FC from the Seurat FindAllMarkers() function and (2) differentially expressed genes between control and injury on a pseudobulk cluster/cell type basis using the Wald statistic. Multiple hypothesis correction was performed at the level of each cluster/cell type and each cluster/cell type was considered a separate family of hypotheses. Pseudobulk analysis was restricted to mouse clusters and cell types that comprised at least ten cells in each sample. Pseudobulk analysis was restricted to human clusters and cell types that comprised at least ten cells in at least four control and at least four TBI samples.

## Supplementary Material



## Data Availability

Code relevant to this manuscript is available at https://github.com/Ciryam-Lab/Lefkowitz-Proteostasis-2026/. The data anlyzed in this article are available on the Gene Expression Omnibus (GEO), accession numbers GSE209552 and GSE269748. Supplementary material is available online [50].

## Theme

One contribution of 20 to a Theo Murphy meeting issue ‘ProteostaSys: a systems view of proteostasis’.
