# Supplementary material for: Traumatic brain injury induces cell-type-specific remodelling of the proteostasis network
Source: Philos Trans R Soc Lond B Biol Sci. 2026 Oct 1;381(1960):20250264. doi: 10.1098/rstb.2025.0264 (PMC13628056; doi:10.1098/rstb.2025.0264)
Supplement: Supplementary file 1 [file rstb.2025.0264.zip › rstb.2025.0264/rstb20250264_si_001.pdf]

Traumatic Brain Injury induces cell-type-specific remodeling of the Proteostasis Network

Jonathan Lefkowitz, Milo Taylor, Aditya Kumar, Ruchira M. Jha, Dhivyaa Rajasundaram, Prajwal Ciryam

Supplementary figures

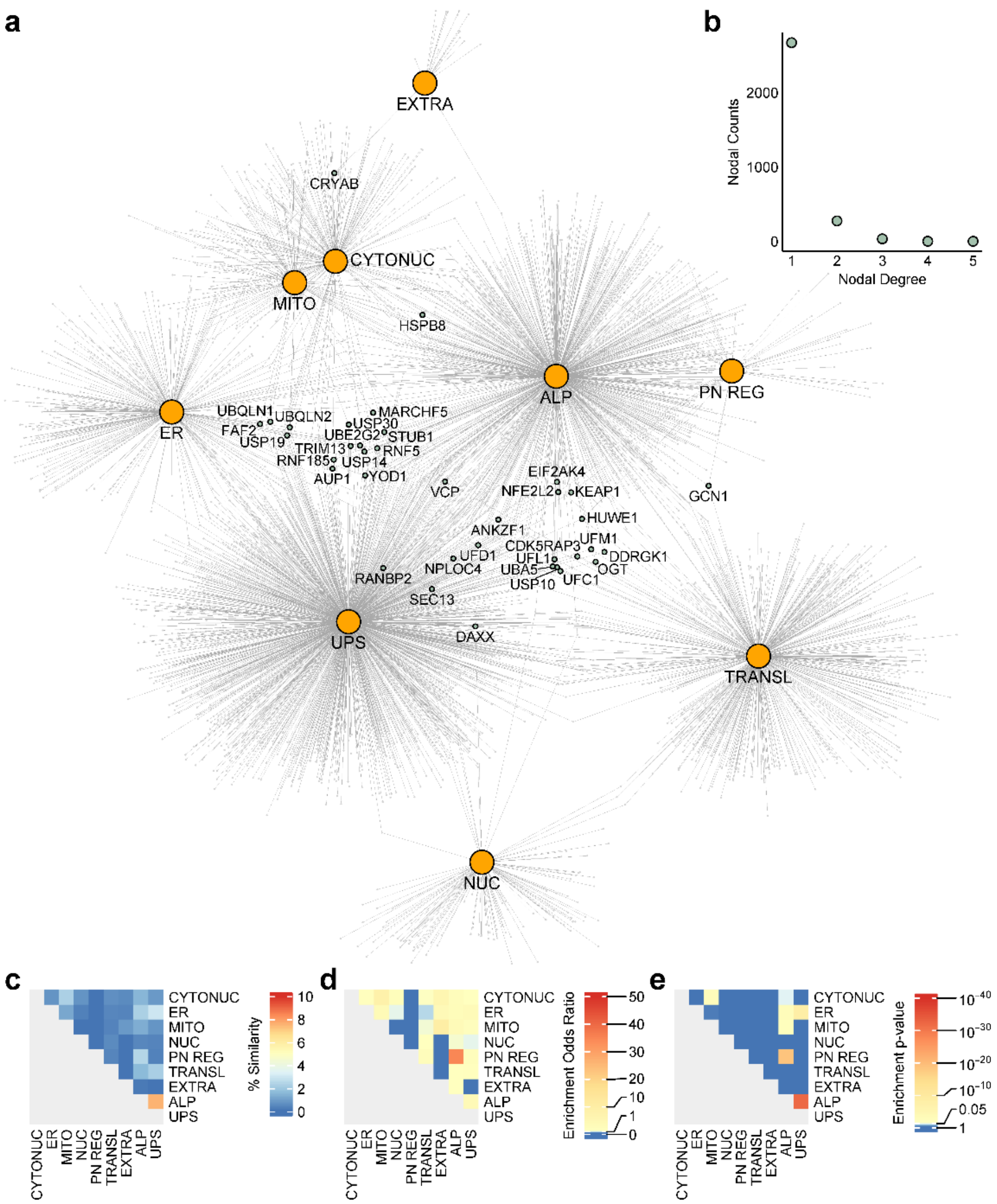

Figure S1. Interrelatedness of Proteostasis Network branches. (a) PN branches shown with genes shared between multiple branches labeled. Other PN genes are shown in light grey. **(b)** Distribution of nodal degrees in PN network based on connections between branches. Pathway interrelatedness is shown based on **(c)** Jaccard similarity (as a percentage), **(d)** Fisher's Exact Test Odds Ratio, and **(e)** Fisher's Exact Test p-value (Holm-Bonferroni adjusted).

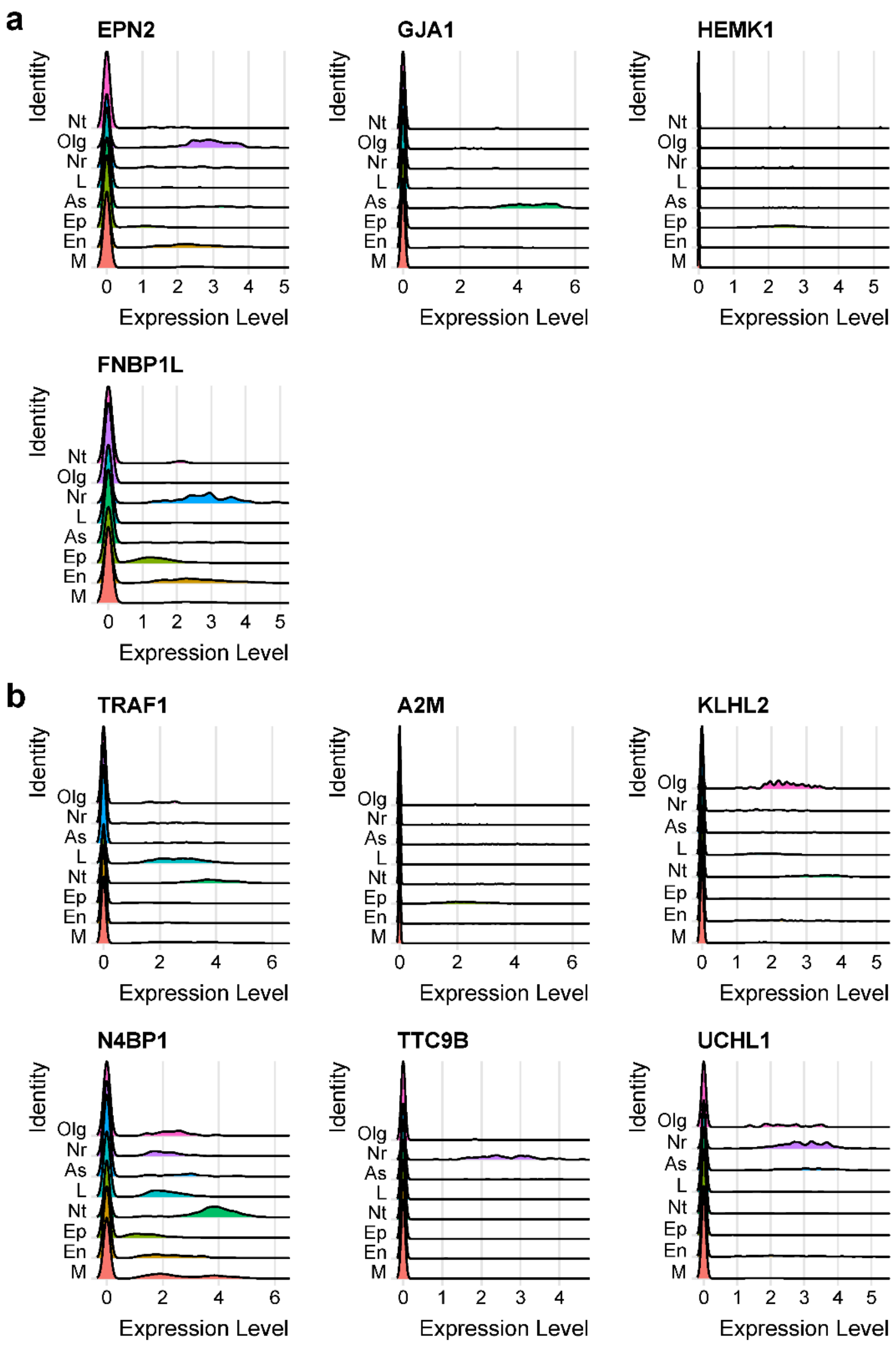

Figure S2. Expression of mouse unique naïve and CCI markers. Example ridge plots showing distribution of cell-type specific expression in cells derived from **(a)** naïve or **(b)** CCI tissue.

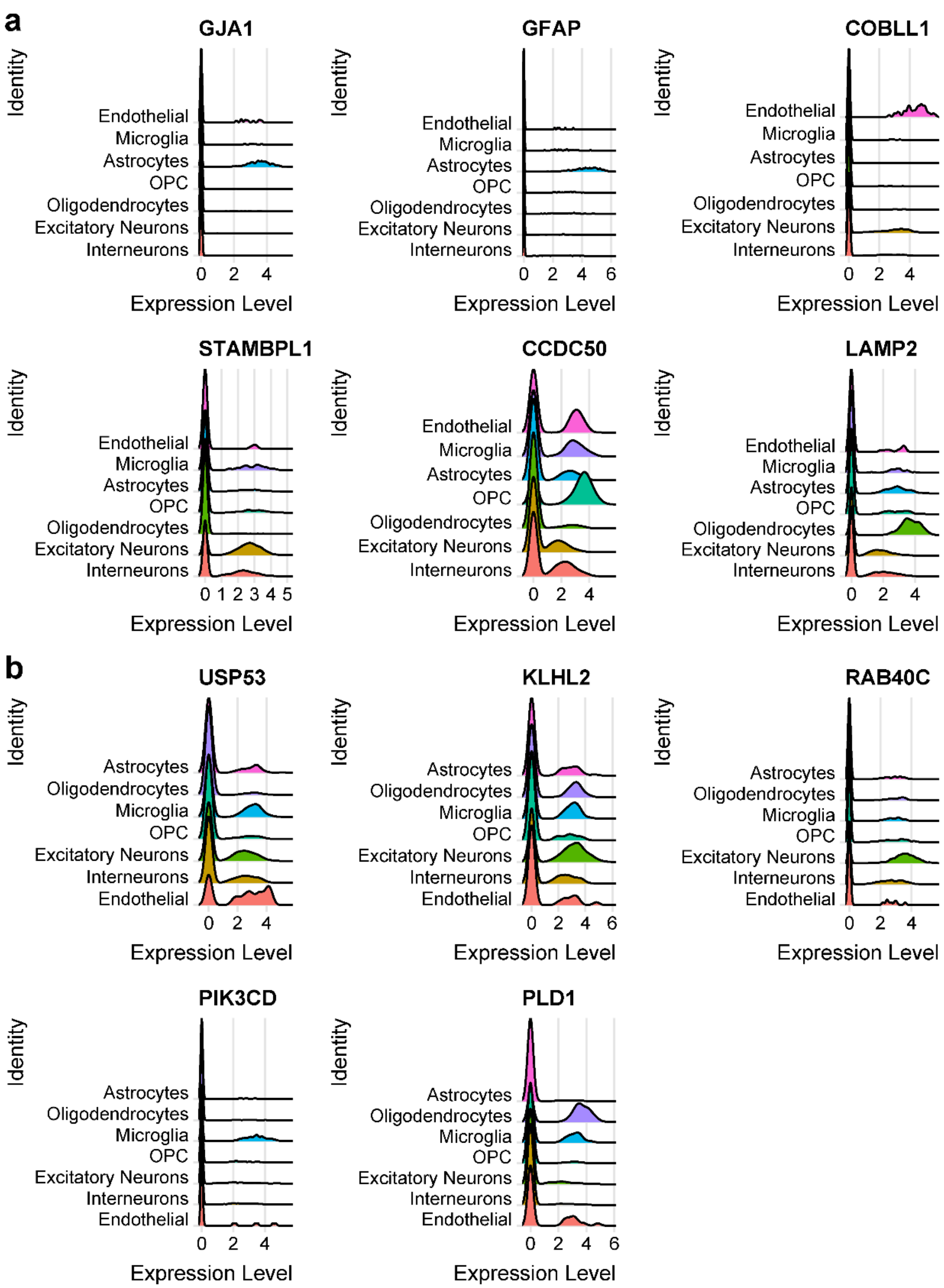

Figure S3. Expression of human unique control and TBI markers. Example ridge plots showing distribution of cell-type specific expression in cells derived from **(a)** control or **(b)** TBI tissue.

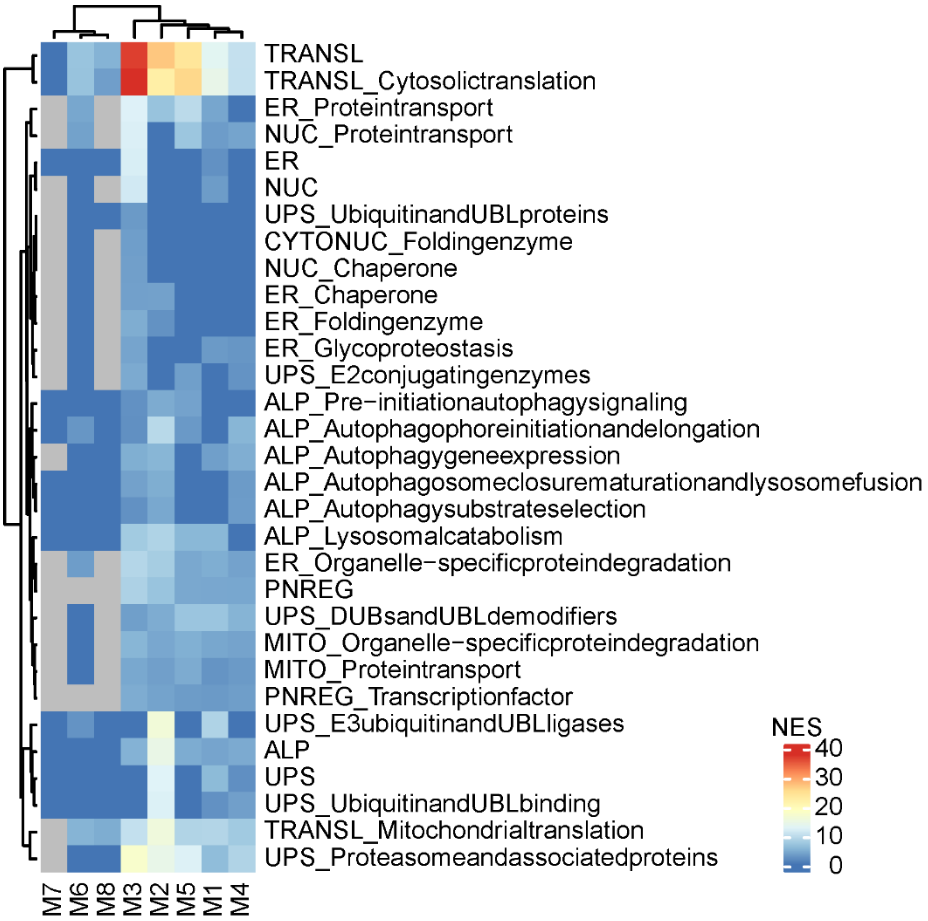

Figure S4. Cell-type specific expression of the Proteostasis Network in mouse microglial subtypes. Normalized Enrichment Score (NES) for each cluster derived from Gene Set Enrichment Analysis (GSEA) using fgsea (positive enrichment only). Dark blue boxes denote NES=0 or fgsea $p_{adj} \geq 0.05$.
